# Enzymatic studies on aromatic prenyltransferases

**DOI:** 10.1007/s11418-020-01393-x

**Published:** 2020-03-17

**Authors:** Takahiro Mori

**Affiliations:** Graduate School of Pharmaceutical Sciences, The University of Tokyo, 7-3-1 Hongo, Bunkyo-ku, Tokyo, 113-0033 Japan

**Keywords:** Biosynthesis, Enzyme engineering, Aromatic prenyltransferase, Prenylated compounds

## Abstract

Aromatic prenyltransferases (PTases), including ABBA-type and dimethylallyl tryptophan synthase (DMATS)-type enzymes from bacteria and fungi, play important role for diversification of the natural products and improvement of the biological activities. For a decade, the characterization of enzymes and enzymatic synthesis of prenylated compounds by using ABBA-type and DMATS-type PTases have been demonstrated. Here, I introduce several examples of the studies on chemoenzymatic synthesis of unnatural prenylated compounds and the enzyme engineering of ABBA-type and DMATS-type PTases.

## Introduction

The prenylated indole alkaloids and prenylated aromatic compounds isolated from plants and microorganisms show broad structural diversity and various biological activities [1–6]. The prenylation may increase the lipophilicity and/or binding ability to target protein that directly influences the biological activity [7, 8]. The prenylation to aromatic compounds is catalyzed by the several enzyme groups of prenyltransferases (PTases), including membrane-embedded UbiA-type, bacterial and fungal ABBA-type, and fungal dimethylallyl tryptophan synthase (DMATS)-type PTases [9–20].

UbiA-type PTases possess a conserved (N/D)DXXD motif for binding of Mg^2+^ ion and diphosphate that is also conserved in the isoprenyl diphosphate synthases [9, 10]. The enzymes in this group are observed in the ubiquinone and menaquinone biosynthesis [10], membrane lipids biosynthesis in archaea [21], in the biosynthesis of prenylated aromatic secondary metabolites in plants [1], and fungal meroterpenoid biosynthesis [22]. On the other hand, ABBA-type and DMATS-type PTases from microorganisms are soluble proteins and do not contain diphosphate and metal ion binding motif [11–18, 20]. The soluble aromatic PTases are involved in the biosynthesis of secondary metabolites in bacteria and fungi.

In the present review, several examples of the recent studies on chemoenzymatic synthesis and the enzyme engineering of soluble ABBA-type and DMATS-type PTases to generate unnatural prenylated aromatic compounds are provided.

## Soluble aromatic PTases

**Fig. 1:**
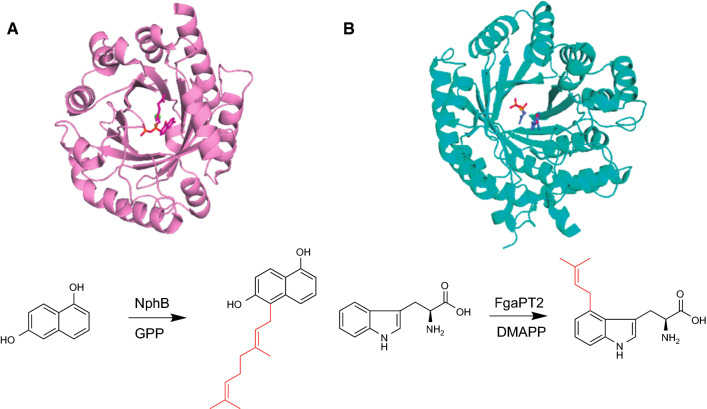
The overall structures of ABBA-type and DMATS-type PTases. The crystal structure and reaction of **a** NphB and **b** FgaPT2

ABBA-type and DMATS-type aromatic PTases catalyze prenylation of dimethylallyl diphosphate (DMAPP) and/or geranyl diphosphate (GPP) to aromatic compounds in bacteria and fungi. The ABBA-type PTases are identified from both of bacteria and fungi, and the CloQ from *Streptomyces roseochromogenes* var. *oscitans* is a first characterized ABBA-type of PTases in 2003, which is involved in the biosynthesis of clorobiocin [23]. Different from membrane-bound UbiA-type PTases, these enzyme reactions, except for NphB, are metal-independent enzymes [14]. The first crystal structure of ABBA-type PTase was solved with NphB in biosynthesis of the naphterpin derivatives [24]. The X-ray crystal structure of NphB showed the characteristic β/α barrel fold with antiparallel strands, which is completely distinct from UbiA-type PTases [25]. This enzyme group was later called as ABBA PTases due to their α-β-β-α PT folds (Fig. 1A) [13].

On the other hand, DMATS-type PTases, identified in fungi, catalyze the prenylation reactions mainly toward indole derivatives, including tryptophan-containing cyclic dipeptides, indole terpenoids, and tryptophan itself [11, 12, 15, 16, 18]. DMATS PTases are also metal-independent enzymes, which do not have aspartate-rich motifs as in the case of ABBA PTases. However, in several cases, the addition of metal ions such as Ca^2+^ and Mg^2+^ enhances their activities [12]. So far, the DMATS enzymes that catalyzed at all positions of the indole ring have been identified (N-1, C-2, C-3, C-4, C-5, C-6, and C-7 prenylation DMATS). The structural analysis of DMATS enzymes revealed that the overall structures share the similar α-β-β-α PT folds as in the case of ABBA-type PTases (Fig. 1b) [26]. In many cases, both of ABBA-type and DMATS-type show broad substrate flexibility towards aromatic substrates [13, 27–41] while these enzymes show narrow specificity toward length of prenyl donors [11, 13, 14, 26–42].

## Chemoenzymatic syntheses of various prenylated compounds

### Specificity for aromatic compounds

**Fig. 2:**
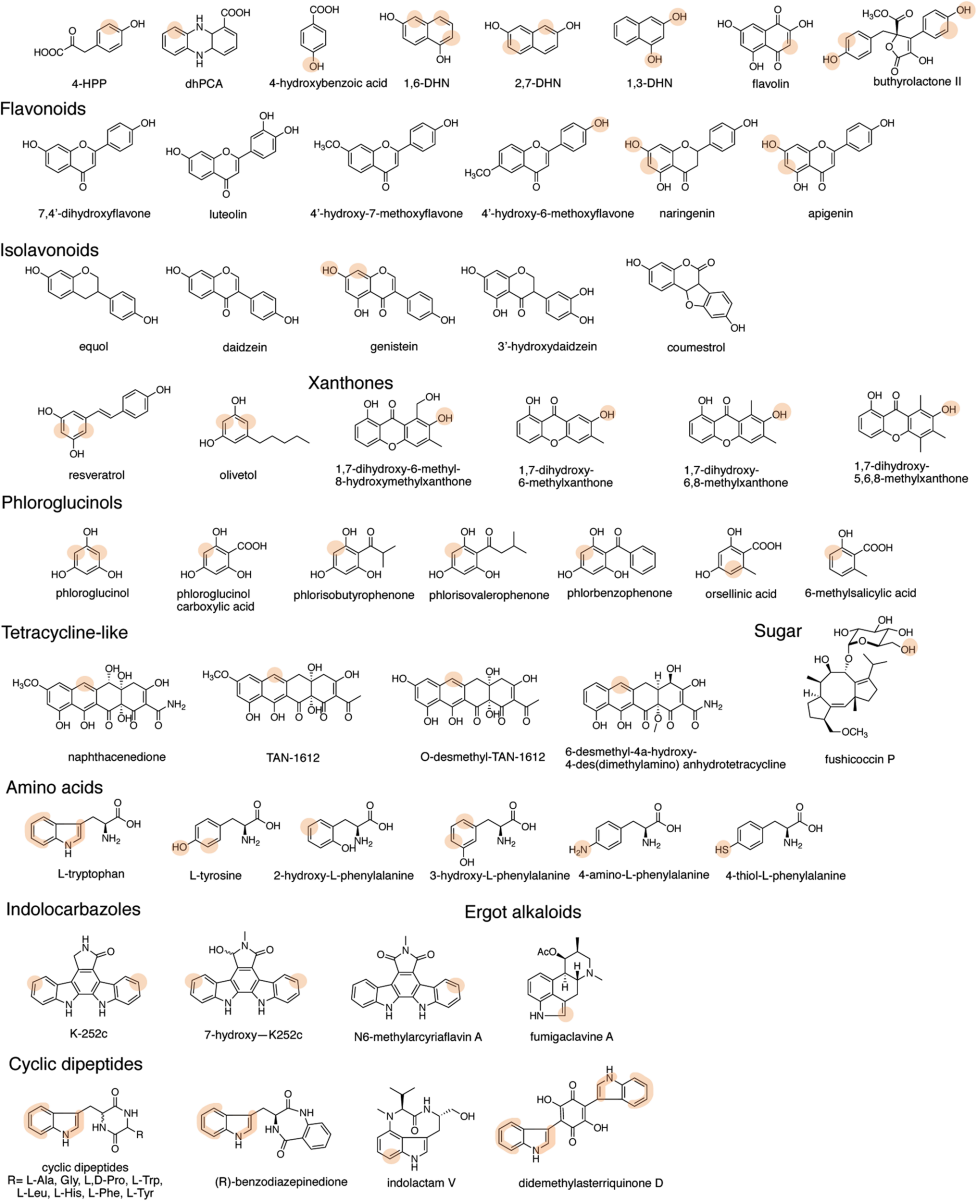
Examples of the prenylation substrates of ABBA-type and DMATS-type PTases. The highlighted atoms are the major prenylation points. The prenylation positions of non-highlighted compounds are not determined

Based on the broad substrate specificity of aromatic prenyltansferases, the chemoenzymatic syntheses of prenylated aromatic derivatives have been performed using the soluble PTases (Table 1). The 4-hydroxyphenylpyruvic acid (4-HPP) derivatives, flavonoids, isoflavonoids, phenylpropanoids, dihydronaphthalenes, and stilbenoids were converted to corresponding dimethylallyl or geranyl group attached products using ABBA-type PTases such as CloQ, NovQ, NphB, SCO7190 and so on [23, 36, 41, 43–46]. The prenylated compounds at different position are obtained using enzymes with different regiospecificity (Fig. 2).

**Table 1:** Examples of prenyltransferases and their substrates

Enzyme	Organism	Prenyl acceptor (examples)	Prenyl donor	References
ABBA-type PTases				
CloQ	*Streptomyces roseochromogenes*	4-HPP, flavonoids, isoflavonoids, stilbenoid	DMAPP	[46]
NovQ	*Streptomyces spheroids*	Phenylpropanoids, flavonoids, DHNs	DMAPP	[45]
NphB	*Streptomyces sp.* CL190	4-HPP, plant polyketides, DHNs	GPP	[24, 38, 47]
Fnq26	*Streptomyces cinnamonensis* DSM 1042	DHNs, flavolin, 4-hydroxybenzoic acid	GPP	[43]
SCO7190	*Streptomyces coelicolor* A3	Plant polyketids, DHNs	DMAPP	[24, 38, 47]
XptB	*Aspergillus nidulans*	Xanthone	DMAPP	[48]
VrtC	*Penicillium aethiapicum*	Tetracycline-like naphthacenediones	DMAPP, GPP	[49]
PaPT	*Phomopsis amygdali*	Fusicoccin P		[50]
TleC	*Streptomyces blastmyceticus*	Indolactam V	DMAPP, GPP, FPP	[83–87]
MpnD	*Marinactinospora thermotolerans*	Indolactam V	DMAPP, GPP, FPP, GGPP, GFPP	[83–87]
AtaPT	*Aspergillus terreus*	Lignanoids, xanthones, quinoline alkaloids, coumarins, benzophenones, curcuminoid, hydroxynaphthalenes	DMAPP, GPP, FPP, GGPP, PPP	[88]
Cyanobactin PTases				
LynF	*Lyngbya aestuarii*	Tyr residue in cyclic peptides, *N*-boc-tyrosine	DMAPP	[51]
PagF	*Oscillatoria agardhii*	L-Tyr, *N*-acetyl-L-Tyr, *N-*boc-L-Tyr, Tyr-Tyr-Tyr, Tyr4 residue in cyclic[INPYLYP]	DMAPP	[59]
TruF		Ser and Thr residues in cyclic peptides	DMAPP	[52, 57, 62]
KgpF	*Microcystis aeruginosa* NIES-88	Trp residue in kawaguchipeptine B, Fmoc-Trp	DMAPP	[58]
TyrPT	*Aspergillus niger*	Tyr and Trp derivatives	DMAPP, alkyl-PPs	[31, 63]
SirD	*Leptosphaeria maculans*	Tyr and Trp derivatives	DMAPP, alkyl-PPs	[29, 31, 41, 63, 92]
DMAT-type PTases				
5-DMAT	*Aspergillus clavatus,* *Streptomyces coelicolor*	Tyr and Trp derivatives, indolocarbazoles	DMAPP, alkyl-PPs	[63, 70, 89]
6-DMAT	*Streptomyces ambofaciens*, *Streptomyces violaceusniger*, *Streptomyces* sp. SN-593	Tyr and Trp derivatives, naphthalene derivatives	DMAPP, GPP, alkyl-PPs	[63, 72, 73]
7-DMAT	*Aspergillus fumigatus,* *Neosartorya sp.*	Tyr and Trp derivative, naphthalene derivatives, acylphloroglucinols, flavonoids	DMAPP, alkyl-PPs	[44, 63, 66]
AnaPT	*Neosartorya fischeri*	Tyr and Trp derivative, Trp-containing cyclic dipeptides, acylphloroglucinols, flavonoids	DMAPP, alkyl-PPs	[44, 67, 82, 91]
FgaPT2	*Aspergillus fumigatus*	Tyr and Trp derivatives, Trp-containing cyclic dipeptides, indolocarbazoles	DMAPP, alkyl-PPs	[26, 36, 42, 63, 90, 93]
CdpC3PT	*Neosartorya fischeri*	Trp-containing cyclic dipeptides, acylphloroglucinols	DMAPP, alkyl-PPs	[67, 77, 91]
CdpNPT	*Aspergillus fumigatus*	Trp-containing cyclic dipeptides, naphthalene derivatives	DMAPP, alkyl-PPs	[37, 67, 79, 91]
BrePT	*Aspergillus versicolor*	Trp-containing cyclic dipeptides	DMAPP, alkyl-PPs	[30, 91]
FtmPT1	*Aspergillus fumigatus*	Trp-containing cyclic dipeptides, indolylbutenone	DMAPP, alkyl-PPs	[27, 28, 91]

First characterized CloQ from *Streptomyces roseochromogenes* was thought to be specific for 4-HPP [23]. However, recent study on the substrate tolerance of CloQ for various phenolic acceptor revealed that the enzyme accepts flavonoids; 7,4′-dihydroflavone, luteolin, 4′-hydroxy-7-methoxyflavone, and 4′-hydroxy-6-methoxyflavone, isoflavonoids; equol, daidzein, genisein, 3′-hydroxydaidzein, and coumestrol, and stilbenoid; resveratrol to produce corresponding dimethylallyl group attached products (less than 10% yield) [46]. Furthermore, the phenylpropanoids; caffeic acid and *p*-coumaric acid, the (iso)flavonoids; naringenin, apigenin, and the dihydronaphthalenes (DHNs); 1,6-DHN and 2,7-DHN were prenylated with DMAPP by NovQ from *Streptomyces spheroids*, ﻿involved in the biosynthesis of novobiocin [45]. Interestingly, the yield of prenylated phenylpropanoids, flavonoids, and DHNs are much better compared to the enzyme reaction of CloQ (15–90% by NovQ). Similarly, NphB, which catalyzes geranylation reaction, also shows a broad substrate tolerance toward aromatic compounds, and this enzyme accepts 4-HPP, plant polyketides, and DHNs, including olivetol, olivetolic acid, resveratrol, apigenin, naringenin, genistein, daidzein, 1,6-DHN, and 2,7-DHN to generate corresponding geranylated products [24, 38, 47]. Fnq26 from *Streptomyces cinnamonensis* DSM 1042 shows slightly different substrate specificity from NphB, whereas Fnq26 shares ~ 40% identity with NphB [43]. Fnq26 catalyzes regular *O*-prenylation and reverse and regular C-prenylation toward flaviolin, 1,3-DHN, and 4-hydroxybenzoic acid. Further, the NphB homologue SCO7190 from *Streptomyces coelicolor* A3 shows similar substrate specificity to NphB and generates dimethylallyl attached naringenin, olivetol, resveratrol, 1,6-DHN, and 2,7-DHN [24, 38, 47].

Some of fungal ABBA superfamily enzymes accept different type of polyketides and terpenoids. For example, the hydroxylated and methylated xanthone compounds are prenylated by XptB from *Aspergillus nidulans* [48]. VrtC from *Penicillium aethiapicum* and its homologs catalyze prenylation of DMAPP and GPP to tetracycline-like naphthacenedione compounds such as phthacenedione, TAN-1612 (2-acetyl-2-decarboxamido-anthrotainin), and 6-desmethyl-4a-hydroxy-4-des-(dimethylamino)anhydrotetracycline [49]. Moreover, PaPT from *Phomopsis amygdali* accept glycosylated terpenoid fusicoccin P to generate an *O*-prenylated compound fusicoccin J in the fusicoccin A biosynthesis [50].

The cyanobactin prenyltransferases LynF, PagF, KgpF, TruF, TyrPT, and SirD form a group of small ABBA-type proteins that catalyze the prenylation of tryptophan, tyrosine, threonine, or serine residues in ribosomally synthesized and post-translationally modified peptides (RiPPs) [50–61]. In these, LynF and PagF also accept *N*-boc-tyrosine and various tyrosine-containing cyclic peptides [51, 59]. Moreover, PagF used linear peptides with different amino acid sequences as substrates. TruF is encoded in the biosynthetic gene cluster of trunkamide and catalyzes the prenylation of DMAPP on serine or threonine residues of core peptide [62]. Although the substrate selectivity of purified enzyme of TruF has not been elucidated, the expression of *tru* biosynthetic genes in *E. coli* created quite large numbers of prenylated peptide derivatives [52, 57]. The tyrosine and tryptophan derivatives, including 4-amino- and 4-thiol-phenylalanine and methyl- or methoxylated tryptophans were accepted by tyrosine *O*-prenyltransferase SirD from *Leptosphaeria maculans*, involved in the biosynthesis of sirodesmin PL, to give *O*-, *C*-, *N*-, and *S*- prenylated compounds [29, 41, 63].

DMATS superfamily enzymes were used for the production of various prenylated indole-containing compounds. The different DMATSs catalyze prenylation toward different position of indole ring. The catalytic reaction of DMATS are well introduced by Winkelblech et al. in 2015 [12]. Using DMATS enzymes, tryptophan derivatives, L-tryptophan-containing cyclic dipeptides, isomers of L-tyrosine, indolocarbazoles, and phenolic molecules were prenylated [26, 29, 41, 63–81]. Some DMATSs also accept aromatic compounds such as acylphloroglucinols and related compounds. AnaPT from *Neosartorya fischeri* and 7-DMATS from *Aspergillus fumigatus*, and CdpC3PT from *Neosartorya fischeri* catalyze prenylation toward phloroglucinol, orsellinic acid, 6-methylsalicylic acid, phloroglucinol carboxylic acid, phlorisobutyrophenone, phlorisovalerophenone, and phlorbenzophenone [82].

### Specificity for prenyl donors

**Fig. 3:**
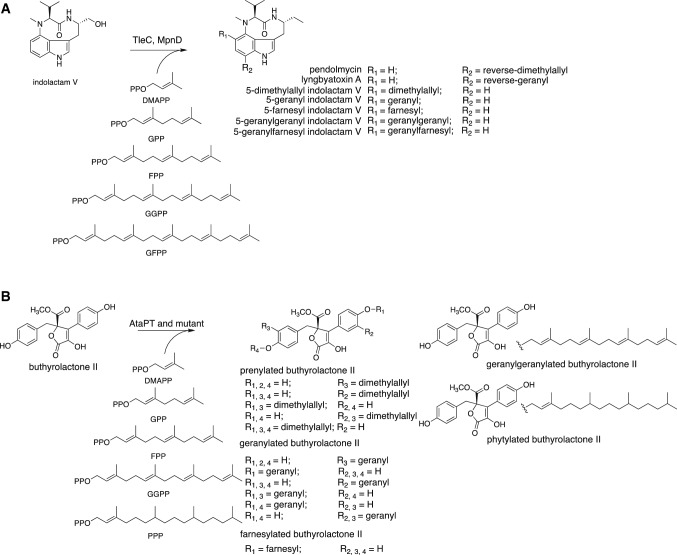
The substrate specificity of **a** TleC, MpnD, and **b** AtaPT toward prenyl donors

In contrast to aforementioned PTases that show broad specificity toward a variety of prenyl acceptors, TleC from *Streptomyces blastmyceticus* and MpnD from *Marinactinospora thermotolerans*, in the biosynthesis of teleocidin and pendolmycin, accept only indolactam V as a prenyl acceptor [83–87]. Instead, these enzymes exhibit broad substrate specificity toward prenyl donors (Fig. 3a). The DMAPP (C5), GPP (C10), farnesyl diphosphate (FPP) (C15), geranylgeranyl diphosphate (GGPP) (C20), and geranylfarnesyl diphosphate (GFPP) (C25) were accepted by the enzymes to generate C-7 position or C-5 position prenylated indolactam V. Moreover, the extremely promiscuous AtaPT from *Aspergillus terreus* was reported to produce 72 prenylated aromatic compounds, including lignanoids, xanthones, quinoline alkaloids, coumarins, benzophenones, curcuminoid, and hydroxynaphthalenes using DMAPP, GPP, and FPP as prenyl donors [88]. The formation of mono-, di-, and/or tri-prenylated compounds were demonstrated. AtaPT also accepts GGPP and phytyl diphosphate (PPP) as prenyl donors to attach geranylgeranyl or phytyl group on (+)-butyrolactone II (Fig. 3b).

**Fig. 4:**
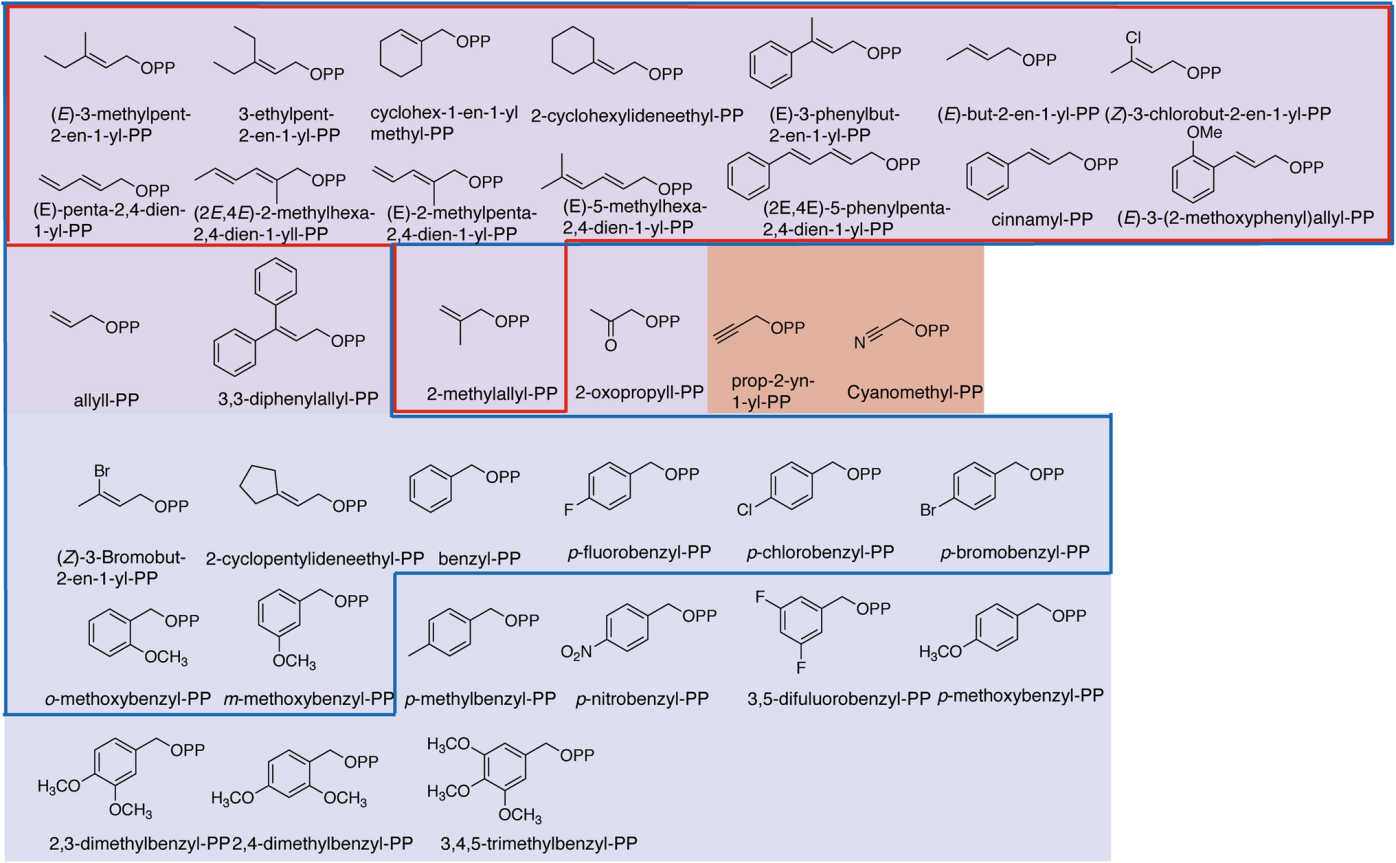
Structures of the synthetic unnatural prenyl donors tested for FgaPT2 and SirD. The substrates highlighted in purple, red, and blue are tested for the both of FgaPT2 and SirD, only SirD, and only FgaPT2, respectively. The substrates enclosed in red and blue frames are accepted by SirD and FgaPT2, respectively

The aromatic PTases can also accept synthetic unnatural alkyl donors, e.g. methylallyl diphosphate (MAPP), 2-pentenyl diphosphate (2-pen-PP), and benzyl diphosphate (benzyl-PP) (Fig. 4). When 2-pen-PP were used as a alkyl donor of FgaPT2 and 5-DMATS, the enzymes generated regular C5-alkylated and C6-alkylated L-tryptophan, respectively [89, 90]. Similarly, the regular C4- and C5-alkylated and regular C5- and C6-alkylated L-tryptophan were produced by FgaPT2 and 5-DMAT, respectively, with MAPP as an allyl donor. Furthermore, C2- and C3- reversely alkylated cyclic dipeptides using MAPP and 2-pen-PP as alkyl donors were delivered by C3-prenyltransferases (CdpC3PT, CdpNPT, and AnaPT) and C2-prenyltransferases (BrePT and FtmPT1) [91]. Interestingly, BrePT and FtmPT1 afforded a mixture of C2- and C3-alkylated diastereomers. C5-, C6-, and C7-benzyl-L-tryptophan derivatives were enzymatically synthesized with 5-DMATS, 6-DMATS, TyrPT, and FgaPT2.

SirD and FgaPT2 were used for further alkyl-diversification of L-Tyr and indole-containing compounds, respectively. Bandari et al. synthesized various alkyl-PP analogues, including allylic, conjugated diene analogues and benzylic substituted substrates shown in Fig. 4 [92, 93]. The 33 unnatural compounds were used as substrates of FgaPT2 and the 20 unnatural compounds were tested for SirD. SirD accepted 15 out of the 20 unnatural alkyl-PP to deliver the corresponding alkylated L-tyrosine. In these, single corresponding *O*-monoalkylated L-tyrosine were mainly produced, while two monoalkylated products were given from cinnamyl-PP and (2*E*,4*E*)-5-phenylpenta-2,4-dien-1-yl-PP. On the other hand, FgaPT2 used 24 out of the 33 synthetic alkyl-PPs. The C4 and C5-regular alkylated and C3 and N1-reversely alkylated L-tryptophan were synthesized through in vitro enzyme reactions. Additionally, C3 position of 7-hydroxy-indolocarbazole was able to be alkylated by FgaPT2 with (*E*)-3-methylpent-2-en-1-yl-PP, 3-ethylpent-2-en-1-yl-PP, cyclohex-1-en-1-ylmethyl-PP, 2-cyclopentylideneethyl-PP, 2-cyclohexylideneethyl-PP (Fig. 4).

### Structure-based engineering of ABBA and DMATS PTases

So far, more than 15 crystal structures of aromatic PTases have been reported [23, 58, 66, 78, 84, 87, 93–102]. The structure-based engineering of PTases were also performed for several enzymes.

The EpzP and PpzP from *Streptomyces cinnamonensis* DSM 104 and *Streptomyces annulatus* 9663, respectively, catalyze prenylation toward phenazine [101]. The crystal structures of EzpP, the docking model with a substrate 5,10-dihydrophenazine-1-carboxylate (dhPCA), and the mutagenesis analysis provided the intimate structural details of the prenylation reaction mechanism. Based on these information together with the sequential comparison between EpzP and PpzP, the catalytic velocity of EpzP was improved by site-directed mutagenesis. V270F mutation was introduced to form π-stacking between dhPCA and Phe residue. As a result, the enzymatic activity of V270F mutant was increased five-times compared to wild type. Furthermore, the substitution of Ala285 with Gln residue to interact with a water molecule in the active site showed ~ 14-fold higher enzymatic activity than wild type.

**Fig. 5:**
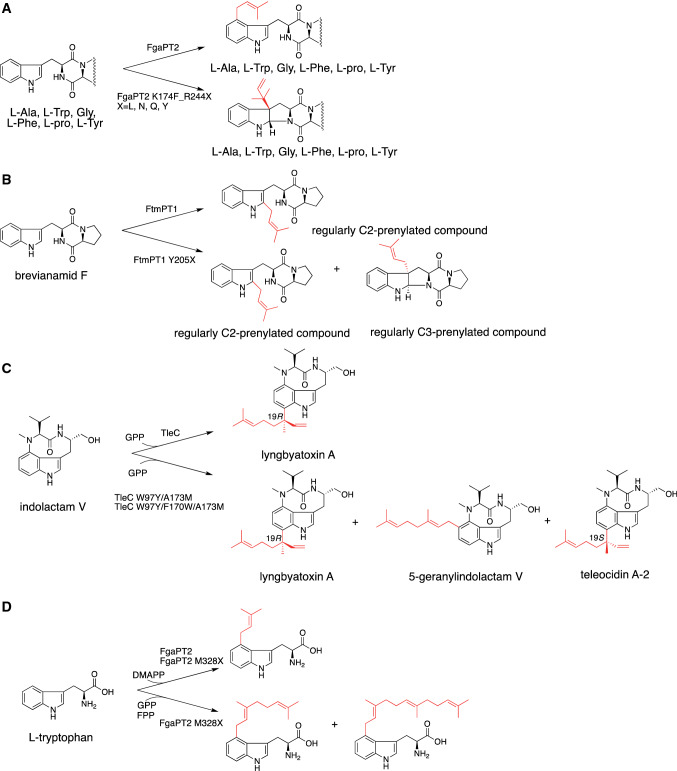
The enzyme reactions of engineered ABBA-type and DMATS-type PTases. The enzyme reaction of **a** the FgaPT2 and its K174F/K244X mutants, **b** the FtmPT1 and its Y205X mutants, **c** the TleC W97Y/A173M and W97Y/F170W/A173M mutants, and **d** FgaPT2 and its M328X mutants

The substrate specificity of FgaPT2 was altered by structure-based mutagenesis experiment. Lys174 residue in FgaPT2, proposed to abstract a proton from prenyl-attached arenium intermediate, was substituted with phenylalanine to stabilize the arenium intermediate and increase the interaction with benzene ring of non-genuine substrate L-tyrosine [26, 103]. The K174F exhibited 4.9-times higher catalytic efficiency toward L-tyrosine than that of wild type, while the activity toward L-tryptophan was almost abolished. Interestingly, the K174F mutant catalyzes C3-prenylation reaction toward L-tyrosine and its analog 4-amino-L-phenylalanine, and *N*-prenylation reaction toward 4-amino-L-phenylalanine as a minor reaction [56]. The specificity for the prenylation of L-tyrosine and L-tryptophan was changed from 1:31 (wild type) to 208:1 (K174F mutant). Furthermore, saturation mutagenesis was performed at Arg244, interacting with carboxylate group of substrates [104]. The prenylation activities of 13 Arg244 mutants toward tryptophan-containing cyclic dipeptides were increased up to 76-times compared to wild type. Interestingly, the preferences for tryptophan-containing cyclic dipeptides, including cyclo-L-Trp-L-Leu, cyclo-L-Trp-D-Pro, cyclo-L-Trp-L-Pro, cyclo-L-Trp-Gly, cyclo-L-Trp-L-Trp, and cyclo-L-Trp-L-Phe of these mutants were also changed. For example, the wild-type, R244A, R244T, and R244Q prefer cyclo-L-Trp-D-Pro and cyclo-L-Trp-L-Leu, while R244G utilizes cyclo-L-Trp-L-Leu and cyclo-L-Trp-L-Trp as preferable substrates. The combination of the K174F and R244X mutations succeeded to alter the regiospecificity of prenylation from C4-regular prenylation to C3-reverse prenylation toward tryptophan-containing cyclic dipeptides (Fig. 5a) [105].

The structure-based engineering of DMATS to alter the product specificity was also achieved using FtmPT1 from *Aspergillus fumigatus* in ﻿fumitremorgins biosynthesis. FtmPT1 originally catalyzes C2 prenylation reaction to bevianamide F (cyclo-L-Trp-L-Pro). The structure analysis of FtmPT1 suggested that Tyr205 residue in FtmPT1 interacts with ketone-group of bevianamide F. The saturation-mutagenesis at Tyr205 revealed that the 15 mutants generate regularly C3-prenylated brevianamide F, but not at C2 position [106]. The substrate specificity analysis of the two selected mutants Y205N and Y205L revealed that these mutants generated C3-reverse prenylated compounds as predominant products when cyclo-D-Trp-D-Pro, cyclo-D-Trp-L-Pro, and cyclo-L-Trp-D-Pro were used as substrates (Fig. 5b).

On the other hand, the engineering of substrate specificity toward prenyl donors was demonstrated using MpnD, and TleC. MpnD and TleC prefer to utilize C5 DMAPP and C10 GPP, respectively, and catalyze attachment of prenyl donor at the C-7 position of indolactam V in a reverse fashion [85]. The structural analysis of MpnD and TleC complexed with substrates suggested that the three amino acid residues Trp97, Phe170, and Ala173 in TleC and Tyr80, Trp157, and Met159 in MpnD regulate the selectivity of the length of prenyl donor and regiospecificity of prenylation position (Fig. 5c). Based on these observations, TleC A173M, TleC W97Y/A173M, and TleC W97Y/F170W/A173M, MpnD M159A were constructed and analyzed. The preference for prenyl donors of TleC A173M switched from GPP to the smaller DMAPP. On the contrary, M159A substitution in MpnD improved the GPP prenylation activity to generate lyngbyatoxin A, while DMAPP prenylation activity was decreased. Moreover, the TleC W97Y/A173M and TleC W97Y/F170W/ A173M mutants newly produced teleocidin A-2, C-19-epimer of lyngbyatoxin A, and 5-geranylindolactam V in addition to lyngbyatoxin A.

Similar manipulation of prenyl donor substrates was also performed using FgaPT2. The structure-based modeling of FgaPT2 with substrate suggested that the side chain of Met328 protrudes toward the active site and would decrease the size of active site. Thus, Met328 was thought to regulate the substrate specificity of the length of prenyl donor. The substitution with smaller side chain, including M328A, M328C, M328S, and M328G significantly increased the activity for GPP and FPP prenylation (Fig. 5d) [107]. Furthermore, the model also suggested that the active site residues Lue263 and Tyr398 could also interfere with terminal isoprene unit of FPP. The large-to-small substitution of Leu263 and Tyr398 with Ala and Phe, respectively, improved the FPP prenylation activity.

## Conclusion

The development of the sequencing technology and the improvement of methodology to characterize the enzymes have accelerated the understanding of the biosynthesis of secondary metabolites. By using these techniques, dozens of ABBA-type and DMATS-type aromatic PTases were functionally and structurally characterized. The accumulation of the knowledge in enzymes provided the chance for the application and engineering of these aromatic PTases. The results presented in this review would be the model cases toward utilization of the secondary metabolite enzymes to generate structurally diversified and biologically active unnatural novel molecular scaffolds for drug discovery.
